# Paraoxonase 1 Phenotype and Mass in South Asian versus Caucasian Renal Transplant Recipients

**DOI:** 10.1155/2012/608580

**Published:** 2012-06-03

**Authors:** Philip W. Connelly, Graham F. Maguire, Michelle M. Nash, Lindita Rapi, Andrew T. Yan, G. V. Ramesh Prasad

**Affiliations:** ^1^Department of Medicine, Keenan Research Centre, Li Ka Shing Knowledge Institute, St. Michael's Hospital, University of Toronto, Toronto, ON, Canada M5B 1W8; ^2^Keenan Research Centre, Li Ka Shing Knowledge Institute, St. Michael's Hospital, Toronto, ON, Canada M5B 1W8; ^3^Division of Cardiology, Keenan Research Centre, Li Ka Shing Knowledge Institute, St. Michael's Hospital, University of Toronto, Canada

## Abstract

South Asian renal transplant recipients have a higher incidence of cardiovascular disease compared with Caucasian renal transplant recipients. We carried out a study to determine whether paraoxonase 1, a novel biomarker for cardiovascular risk, was decreased in South Asian compared with Caucasian renal transplant recipients. Subjects were matched two to one on the basis of age and sex for a total of 129 subjects. Paraoxonase 1 was measured by mass, arylesterase activity, and two-substrate phenotype assay. Comparisons were made by using a matched design. The frequency of PON1 QQ, QR and RR phenotype was 56%, 37%, and 7% for Caucasian subjects versus 35%, 44%, and 21% for South Asian subjects (*χ* ^2^ = 7.72, *P* = 0.02). PON1 mass and arylesterase activity were not significantly different between South Asian and Caucasian subjects. PON1 mass was significantly associated with PON1 phenotype (*P* = 0.0001), HDL cholesterol (*P* = 0.009), LDL cholesterol (*P* = 0.02), and diabetes status (*P* < 0.05). Arylesterase activity was only associated with HDL cholesterol (*P* = 0.003). Thus the frequency of the PON1 RR phenotype was higher and that of the QQ phenotype was lower in South Asian versus Caucasian renal transplant recipients. However, ethnicity was not a significant factor as a determinant of PON1 mass or arylesterase activity, with or without analysis including PON1 phenotype. The two-substrate method for determining PON1 phenotype may be of value for future studies of cardiovascular complications in renal transplant recipients.

## 1. Introduction

 Kidney transplant recipients (KTRs) are known to be at increased risk for cardiovascular disease and by three years after transplant about 40% of KTRs have experienced a cardiovascular disease-related event [1]. The incidence of disease is occurring in the context of best medical practice for treatment of established cardiovascular risk factors. Thus there is a need to identify novel risk factors with the objective of improving treatment and reduction of the incidence of cardiovascular disease. 

 End-stage renal disease, the precursor to KTR, is increasing in individuals of South Asian ethnicity relative to subjects of Caucasian ethnicity [1]. There is a need to characterize these subjects and determine whether novel risk factors for cardiovascular disease are present in an ethnicity-dependent manner. The current study was designed to compare two novel risk factors, adiponectin and paraoxonase 1 (PON1), as candidates for differing risk between South Asian and Caucasian KTR. We observed significantly lower total and high molecular weight adiponectin in South Asian KTR [2]. Stage 3 chronic renal disease, defined as cystatin C-based estimated glomerular filtration rate (eGFR) less than 60, was associated with lower PON1 mass in subjects with diabetes [3]. In this paper we present the analysis for PON1 and report that PON1 Gln192Arg (Q192R) phenotype is different by ethnicity. 

## 2. Methods

### 2.1. Patient Selection

 Subjects were recruited with age (±10 years) and gender matched in a 1 : 2, South Asian : Caucasian ratio. Inclusion criteria were KTR of stated ethnicity with stable renal function (less than 20% fluctuation of creatinine in the 2 months preceding recruitment), within 3 to 60 months of transplant, and able to provide informed consent. Subjects were excluded if ethnicity was uncertain, if they were multiorgan transplant recipients, if they had unstable renal function, or if they were unable to understand the consent process. Subjects who consented provided a blood sample at the time of clinic visit in the nonfasting state. The study was approved by the Human Research Ethics Board of St. Michael's Hospital. A total of 60 Caucasian males, 30 South Asian males, 26 Caucasian females, and 13 South Asian females were studied, slightly exceeding the study design target sample of 120 subjects. A complete description of the subjects can be found in Prasad et al. [2].

### 2.2. Measurements

 Routine clinical laboratory measurements were performed for standard blood clinical chemistry analytes, including creatinine, glucose, and hemoglobin A1c. Body mass index was determined from weight and height. Clinical records were used for determination of the presence of diabetes according to the 2008 Canadian Diabetes Association guidelines. Lipoproteins were analyzed by ultracentrifugation according to the Lipid Research Clinics protocol. High-density lipoprotein cholesterol was determined by a homogeneous assay on the Beckman LX-20 instrument (Beckman, Mississauga, ON). Cystatin C, apolipoprotein B, and apolipoprotein AI were measured using the Dade-Behring BN Prospec (Dade-Behring, Mississauga, ON).

 PON1 mass was measured on serum samples by Western blot with a recombinant standard and calibrated with a normal serum sample [4]. PON1 arylesterase activity was measured using 3 mM phenyl acetate to determine the arylesterase activity and expressed in U/mL. PON1 phenotype was determined using the two-substrate assay of Richter et al. [5] Data for the hydrolysis of 4-(chloromethyl)phenyl acetate (CMPA) was plotted on the *x*-axis and data for hydrolysis of phenyl acetate in the presence of 2 M NaCl (high salt conditions) was plotted on the *y*-axis. Data was transformed using the arctangent of the ratio of CMPA/arylesterase activity to derive the radians from the origin. For convenience in presentation, these values were converted to degrees. The activity for purified PON1 Q192 and PON1 R192 was used to confirm identity of the QQ and RR samples. The PON1 phenotypes were assigned as follows: QQ, arctangent range from 11.8 to 17.08 degrees; QR, arctangent range from 20.7 to 34.9 degrees; and RR, arctangent range from 51.3 to 57.4 degrees.

### 2.3. Statistical Analyses

 Analyses were carried using SAS 9.3. Analysis of variance with the matched design was performed using Proc Mixed. A *P* value < 0.05 was used for statistical testing.

## 3. Results

 Demographic data and biochemical data are summarized in Table 1. Subjects were well matched by age at transplant (50 versus 52 years for South Asian versus Caucasian) and time after transplant (65 versus 71 months for South Asian versus Caucasian). None of the established risk factors were significantly different between South Asian and Caucasian subjects by univariate analysis. PON1 mass and arylesterase activity were similar between the two groups and had a similar distribution as seen by comparison of the 25th and 75th percentiles.

 Univariate Spearman correlation was determined for PON1 mass and arylesterase activity by gender. Among women, arylesterase activity correlated with PON1 mass (*r* = 0.71, *P* < 0.0001), HDL cholesterol (*r* = 0.45, *P* = 0.004), and apoAI (*r* = 0.35, *P* = 0.03). Among men, arylesterase activity correlated with PON1 mass (*r* = 0.78, *P* < 0.0001), but not with HDL cholesterol (*r* = 0.14, *P* = NS) and weakly with apoAI (*r* = 0.21, *P* = 0.05). PON1 mass was not significantly correlated with either HDL cholesterol or apoAI in either gender.

**Figure 1:**
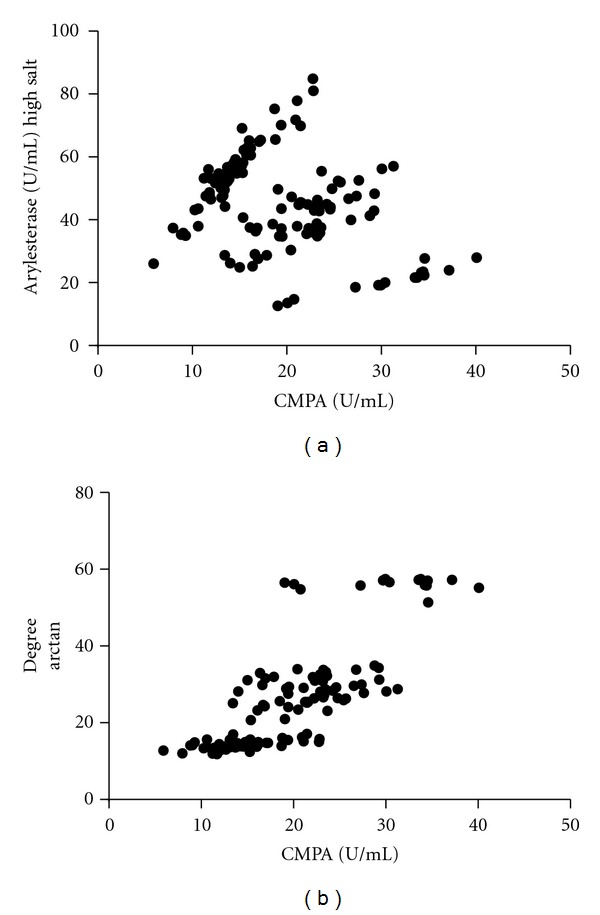
(a) Activity measured using phenyl acetate at high salt conditions versus CMPA. (b) Transformation of activities using arctangent (*x*/*y*) ∗ 180/3.14 versus CMPA. This transformation converts *x* and *y* coordinates into the angle in degrees.

PON1 phenotype was determined as described by Richter et al. [5] using the two-substrate method. We observed clear separation of the results into three phenotypes representing the QQ, QR, and RR phenotypes (Figure 1). The frequency of QQ, QR, and RR was significantly different (*χ* ^2^ = 7.72, *P* = 0.02) between South Asian and Caucasian subjects with the RR phenotype being 3 times more prevalent in South Asian subjects (Table 2). 

 The determinants of PON1 mass were determined with ethnicity and the covariates PON1 phenotype, lipoprotein concentrations, diabetes status, and renal status as covariates. In spite of the difference in PON1 phenotype, ethnicity was not a significant determinant of PON1 mass. In contrast, PON1 phenotype, diabetes status, HDL cholesterol, and LDL cholesterol were all significantly associated with PON1 mass (Table 3).

 Next the determinants of serum arylesterase were studied (Table 4). Again, ethnicity was not significantly associated with arylesterase activity. Among the variables tested, only HDL cholesterol was significantly associated with arylesterase activity (*P* = 0.003). When regression analysis was performed including PON1 mass as an independent variable, PON1 mass had a partial *r* ^2^ of 0.67 (*P* < 0.001), PON1 phenotype had a partial *r* ^2^ of 0.11 (*P* < 0.001), and HDL cholesterol had a partial *r* ^2^ of 0.007 (*P* = 0.05). Thus 77% of the variation on arylesterase activity was accounted for by PON1 mass and phenotype and less than 1% was accounted for by HDL cholesterol.

 Neither creatinine-based (MDRD) nor cystatin C-based (Arnal-Dade) estimates of glomerular filtration rate (eGFR) showed a relationship with PON1 mass or arylesterase activity (data not shown).

## 4. Discussion

 PON1 has been extensively studied as a risk factor for cardiovascular disease independent of lipoprotein concentrations [6]. Differences in PON1 between subjects have been shown to be due to genetic polymorphisms [7, 8], presence of diabetes [9], presence of hepatic disease [10], and presence of renal disease [3, 11–15]. The complexity of the genetic polymorphism at the PON1 locus may confound studies of PON1 and thus it has been suggested that determination of the PON1 phenotype, mass, and activity be measured [5, 16]. We report that the phenotypic prevalence of the QR and RR phenotype is higher in South Asian compared with Caucasian KTR subjects. The frequency of the phenotypes in Caucasians is similar to that reported for renal transplant patients in Debrecen, Hungary [17, 18]. The frequency of the phenotypes in South Asians is consistent with recently published data on the frequency of the of Gln192Arg polymorphism in North-West Indian Punjabis [6]. It is also similar to that reported for patients with CAD or T2DM from Hyderabad, India [19]. Interestingly, the frequency of the RR was 11.7% in control subjects from Hyderabad, similar to the frequency we observed for Caucasians.

 In contrast to the difference in phenotype by ethnicity, there was no difference in the mass or arylesterase activity of PON1 by ethnicity in KTR. Further, there was a difference in PON1 mass, but not arylesterase activity by diabetes status. Analysis of indices of diabetes (glucose, hemoglobin A1c) did not show significant associations with PON1 mass or arylesterase activity (data not shown). Post hoc analyses for an interaction between ethnicity and diabetes status were also negative. Thus, in this group of KTR subjects, diabetes was a limited determinant of PON1 status. Similarly, an a priori hypothesis was that renal function would be a determinant of PON1. However, neither creatinine nor cystatin C-based estimates of glomerular filtration rate were associated with PON1. This may be due to other factors in KTR being primary determinants of PON1. It should also be noted that the study design was to restrict subjects to a range of post renal transplant from 3 to 60 months. Thus although most of the subjects with diabetes had pre-transplant diabetes, the duration of posttransplant factors was limited to the relatively early time points. Most previous studies of non-KTR have been of subjects with an average duration of diabetes greater than 15 years [20, 21]. Thus one explanation for the absence of associations with diabetes and eGFR could be the shorter duration after transplant in this study.

 PON1 mass, but not arylesterase activity, has been reported to be inversely correlated with mortality in patients on dialysis [22]. In contrast, PON1 genotypes have not been associated with increased cardiovascular risk in renal transplant recipients [23]. Future studies will require measurement of PON1 phenotype by enzymatic activity and PON1 concentration in order to fully evaluate the predictive value of PON1 as a cardiovascular risk factor in kidney transplant recipients.

## Figures and Tables

**Table 1:** 

Parameter	All patients		
	South Asian (*N* = 43)	Caucasian (*N* = 86)	*P*-value
Age at transplant (years)	48 ± 11	50 ± 11	NS
Age at study visit (years)	50 ± 10	52 ± 11	NS
Gender (M/F)	30/13	60/26	NS
Time post transplant (months)	65 ± 47	71 ± 48	NS
BMI (kg/m^2^)	26.9 ± 4.1	28.0 ± 6.0	NS
Total cholesterol (mmol/L)	4.8 ± 1.6	4.4 ± 1.1	NS
HDL cholesterol (mmol/L)	1.1 ± 0.4	1.1 ± 0.3	NS
LDL cholesterol (mmol/L)	3.1 ± 1.2	2.7 ± 0.9	0.07
Triglycerides (mmol/L)	1.8 ± 0.9	1.6 ± 0.7	NS
VLDL cholesterol	0.5 ± 0.4	0.5 ± 0.3	NS
VLDL triglycerides	1.3 ± 0.8	1.2 ± 0.6	NS
ApoAI (mg/L)	1.4 ± 0.3	1.4 ± 0.3	NS
ApoB (mg/L)	0.9 ± 0.3	0.8 ± 0.2	NS
eGFR by MDRD (mL/min/1.73 m^2^)	66 ± 21	60 ± 19	NS
Cystatin C (mg/L)	1.2 ± 0.4	1.2 ± 0.3	NS
eGFR by Cystatin C (mL/min/1.73 m^2^)	65 ± 22	62 ± 20	NS
C-reactive protein (mg/L)	4.5 ± 7.9	6.0 ± 13	NS
PON1 (*μ*g/mL) median (quartiles)	100 (84, 122)	105 (81.7, 127)	NS
PON1 arylesterase (U/mL) median (quartiles)	79.5 (72.7, 90.4)	81.4 (72.2, 91.8)	NS
PON1 CMPA activity (U/mL) median (quartiles)	19.4 (15.3, 26.8)	16.5 (13.6, 22.8)	0.08
PON1 arylesterase activity salt stimulated (U/mL) median (quartiles)	45.4 (35.0, 54.7)	47.8 (37.3, 56.7)	NS

**Table 2:** Frequency of PON1 phenotypes by ethnicity.

Ethnicity	PON1 QQ (*n*, %)	PON1 QR (*n*, %)	PON1 RR (*n*, %)
Caucasian	48 (55.8%)	32 (37.2%)	6 (7%)
South Asian	15 (34.9%)	19 (44.2%)	9 (20.9%)

**Table 3:** Mixed model ANOVA analysis with PON1 mass as the dependent variable.

Variable	*P* value
Ethnicity	NS
PON1 phenotype	0.0001
Diabetes	<0.05
HDL cholesterol	0.009
LDL cholesterol	0.02

**Table 4:** Mixed model ANOVA analysis with arylesterase activity as the dependent variable.

Variable	*P* value
Ethnicity	NS
PON1 phenotype	NS
Diabetes	NS
HDL cholesterol	0.003
LDL cholesterol	NS
